# Supplementary material for: Are carbon emissions trading and green financial instruments synergistic? -Comprehensive quantitative research based on content analysis
Source: PLoS One. 2024 Mar 7;19(3):e0298601. doi: 10.1371/journal.pone.0298601 (PMC10919601; doi:10.1371/journal.pone.0298601)
Supplement: S1 Data — (ZIP) [file pone.0298601.s001.zip › Data/地级市Z_F工作报告.zip!/╦─┤¿/π≥╓▌╩╨/└┤╫╘╣1⁄2╓┌║┼í╛┬φ┐╦╩2╛▌═°í┐_2017.docx]

各位代表：

现在，我代表市人民政府，向大会报告工作，请予审查，并请市政协委员和列席人员提出意见。

过去五年工作回顾

2012年以来的五年，是我市抢抓机遇、开拓奋进的五年。五年来，在省委、省政府和市委的坚强领导下，在市人大、市政协的监督支持下，我们紧紧依靠全市人民，主动适应新常态，主动作为稳增长，锐意进取、攻坚克难，全市经济平稳较快发展，社会保持和谐稳定。预计2016年，全市完成地区生产总值1478亿元，年均增长11.4%，连跨五个百亿元台阶，总量由全省第9位上升到第6位；规模以上工业增加值年均增长13.3%；全社会固定资产投资1730亿元，年均增长28.4%，总量由全省第10位上升到第2位；社会消费品零售总额636亿元，年均增长14.6%；地方一般公共预算收入136.5亿元，年均增长15.8%，总量由全省第5位上升到第2位；城镇居民人均可支配收入28975元，年均增长10.7%；农村居民人均可支配收入12529元，年均增长12.1%。主要经济指标连续五年全省领先，增幅均高于全国、全省平均水平。成功创建国家高新区、国家现代农业示范区、国家园林城市、全国质量强市示范城市，全民预防保健服务体系建设走在全国前列。全市综合实力迈上新台阶，谱写了泸州经济社会发展崭新篇章。

五年来，项目建设和投资拉动成效显著。累计实施重点项目1356个，完成投资4145亿元，是上一个五年的5.8倍。其中，重大产业类项目538个，完成投资1573.7亿元；重大基础设施类项目506个，完成投资1656.3亿元；重大民生和社会事业类项目268个，完成投资865.7亿元；重大生态环保类项目44个，完成投资49.3亿元。民间投资达4080.5亿元，占全社会固定资产投资的69%。加强政府与社会资本合作，实施PPP项目16个，总投资289亿元。组建12家市级投融资公司，资产规模达1057亿元。设立3支投资发展基金，基金规模达230亿元。累计保障项目建设用地14.2万亩，工业用电量、工业用气量、成品油供应量年均分别增长6.6%、12%、12.4%。投资规模不断扩大、结构不断优化、效益不断提升，投资对经济增长的贡献率达65.5%。

五年来，产业发展和转型升级取得重要进展。传统产业不断优化提升，泸州老窖、郎酒等重点酒类企业实现恢复性增长，泸天化改革脱困取得阶段性成效，“三长”完成退城入园，“四大产业”增加值年均增长13%。新兴产业加快培育发展，智能电网、大数据、新能源新材料产业从无到有，高端装备制造、现代医药产业不断壮大，华为公司、步长制药、许继集团等一批知名企业入驻泸州。现代服务业蓬勃发展，西南商贸城（一期、二期）、华润万象汇建成运营，成功创建国家电子商务示范基地，引进7家市外银行机构，创建7个国家4A级景区，旅游总收入年均增长33%。开发建筑业平稳健康发展，建筑业总产值年均增长16.1%。现代农业加快发展，粮食生产持续稳定，“八大特色农业产业”初具规模，建成黄桷坝水库、倒流河水库、锁口水库等一批骨干水利枢纽工程，山东寿光蔬菜产业集团、广东温氏集团等龙头企业入驻泸州。按照“一区多园、一园多点”模式，加快建设“三大园区”，建成区面积由44.1平方公里增加到78.5平方公里。全市产业园区主营业务收入达到1950亿元，年均增长19.6%。

五年来，区域中心城市建设实现重大突破。成功争取首批国家新型城镇化综合试点，进入国家“双两百”规划，全市城镇化率达47.5%。改造提升老城区，加快推进“两江新城”建设，中心城区建成区面积由101.1平方公里增加到135平方公里，人口由101万人增加到136.2万人。大力提升城市品质，不断完善城市功能。93公里绕城高速闭合运行，8个进出口通道建成通车，5座跨江大桥加快建设，打造高品质城市滨江路15公里，新建城市公园23个，改造棚户区5.7万户。教育、文化、卫生、体育等公共服务设施不断完善，区域性商贸物流中心、教育培训中心、医疗健康中心逐步形成，列入首批全国健康城市建设试点城市。理顺城市管理体制，加强城乡环境综合治理，国家卫生城市创建成果进一步巩固。泸县、合江、叙永、古蔺四个县城规模不断扩大，人居环境进一步改善，泸县、合江成功创建为国家卫生县城。纳溪大渡口镇、合江九支镇列入全国首批建制镇示范试点，全市万人以上小城镇达12个，建成幸福美丽新村250个。

五年来，区域性综合交通枢纽加快形成。列入全国重要区域性综合交通枢纽（节点城市）、全国综合运输服务示范城市。建成5条高速公路，通车里程从74.8公里增加到455公里，居全省第3位、川南第1位，实现高速公路县县通。川南城际铁路自贡至泸州段、隆黄铁路叙永至镇雄段开工建设。泸州机场迁建工程加快推进。纳福港城大道、泸合产城大道建成通车，新改建国省干线公路470公里、农村公路5400公里，建制村通达率100%。城北公交枢纽站全面竣工，泸州客运中心站建成运行。泸州开放口岸列入《国家口岸发展“十三五”规划》，泸州港保税物流中心（B型）、国家进境粮食指定口岸建成投运，新开通3条近洋航线、6条铁水联运班列，集装箱年吞吐量从10.1万标箱增加到48万标箱。泸州机场航线由6条增加到18条，旅客年吞吐量从28.5万人次增加到98.5万人次，成为四川第三大航空港。

五年来，改革开放和创新发展深入推进。扎实推进重点领域改革，实施改革事项138项。深入推进行政审批制度改革，全面公布市、县两级行政许可事项目录。在全国率先开展不动产统一登记、农村土地制度改革三项试点、工矿废弃地复垦利用试点。财税体制、投融资体制改革和国企改革取得明显成效。“营改增”试点全面推开。“先照后证”“五证合一、一照一码”等商事制度改革有序推进。开放合作水平全面提升，引进华为、恒大、中冶、万达、碧桂园等世界500强、中国500强企业41家。与韩国平昌郡、武汉市、南京市等一批国际国内城市建立友好合作关系，全方位、多层次、宽领域开放合作格局初步形成。创新驱动能力明显增强，新增国家高新技术企业44家、省级创新型企业36家，高新技术产值从63.1亿元增加到200亿元，年均增长25.9%。

五年来，民生和社会事业全面进步。民生支出占一般公共预算支出的比重达70.3%，一大批涉及群众切身利益的民生难题得到较好解决。脱贫攻坚取得阶段性成效，累计脱贫39.6万人，在全省率先实行医疗扶贫“四个100%”，完成易地扶贫搬迁3.3万人、农村危房改造8.1万户、农网改造2.4万公里，建设“渡改桥”25座。教育事业加快发展，泸州医学院更名为西南医科大学，医教园区初具规模，新改扩建中小学校317所、公办幼儿园108所。4个县区教育均衡发展通过省级评估验收，大班额现象得到有效消除。全民预防保健服务体系建设全面开展，基层医疗卫生服务能力进一步增强。深入开展国家食品安全城市创建工作，食品药品监管切实加强。社会保障水平显著提升，累计新增城镇就业19.8万人，城乡居民养老、医疗保险合并实施，城乡低保应保尽保，列入全国首批养老服务业综合改革试点城市。文体广电和新闻出版事业蓬勃发展，全国文明城市创建加快推进。生态环境持续改善，扎实推进大气、水、土壤污染防治，环境质量和总量减排完成省定目标；深入开展天然林保护、退耕还林、石漠化综合治理，完成全国首批水生态文明城市建设试点工作。社会治理持续加强，社会治安立体化防控体系不断健全，“阳光信访、法治信访、责任信访”全面推进，“六五”普法圆满完成。严格落实安全生产责任制，安全生产形势持续向好。

国防动员、人民防空、民兵预备役建设不断加强，全国双拥模范城创建实现“七连冠”；青年、妇女儿童、老龄、残疾人事业取得新进步，民族、宗教、外事、侨务、对台、统计、消防、保密、气象、档案、地方志等工作取得新成效。

各位代表，五年来，我们深入开展党的群众路线教育实践活动、“三严三实”专题教育、“两学一做”学习教育，扎实推进法治政府、效能政府、廉洁政府建设，自觉接受市人大、市政协监督，严格执行市人大决议、决定，办理人大代表建议550件、政协委员提案1271件，办结率100%。全面加强党风廉政建设，强化行政监察和审计监督，严厉查处政府系统违纪违法案件982起，始终保持惩治腐败高压态势。

各位代表，刚刚过去的2016年，是第七届市政府任期的最后一年，是“十三五”规划的开局之年。预计全市地区生产总值比2015年（下同）增长9.2%，规模以上工业增加值增长11.2%，全社会固定资产投资增长18.2%，社会消费品零售总额增长13.7%，地方一般公共预算收入增长9%，城镇居民人均可支配收入增长8.7%，农村居民人均可支配收入增长10.3%。市七届人大六次会议确定的目标任务较好完成，向全市人民承诺的民生实事全部兑现！

各位代表，过去的五年，是全市人民凝心聚力、专注发展，推动经济实力大提升的五年；是全市人民广泛参与、共建共享，推动城市面貌大改变的五年；是全市人民苦干实干、合力攻坚，推动各项事业大发展的五年。这些成绩的取得，是省委、省政府和市委正确领导的结果，是市人大、市政协监督支持的结果，是广大干部群众团结拼搏的结果。在此，我代表市人民政府，向关心、支持、推动泸州发展的全市人民，向各民主党派、工商联、人民团体，向驻泸部队指战员、武警消防官兵、政法干警，表示衷心的感谢，并致以崇高的敬意！

在肯定成绩的同时，我们也清醒地看到，经济社会发展中还存在许多困难和问题。一是经济总量小、人均低，发展不够、发展不足的问题仍然突出；二是产业结构不优，传统产业转型升级仍需加快，新兴产业亟待培育壮大；三是脱贫攻坚任务十分艰巨，就业、住房、教育、医疗、养老等民生事业与群众期盼还有不小差距；四是社会治理面临新挑战，一些深层次矛盾和问题逐步显现；五是政府自身建设还存在薄弱环节，一些干部能力素质、工作作风还不适应新形势的要求。对此，我们一定高度重视，在今后工作中切实加以解决。

未来五年的主要目标任务

当前和今后一段时期，泸州处于工业化城镇化的加速期、深化改革的攻坚期、全面建成小康社会的决胜期。经济社会发展既有许多有利条件，又面临诸多困难和挑战，我们必须增强忧患意识、责任意识、机遇意识，牢固树立和贯彻新发展理念，认真落实省委、省政府和市委决策部署，围绕“三个走在前列、三个显著提升”，着力“三抓三主动”，扎实推进“六个突破”，奋力实现“决胜全面小康、建成区域中心”的宏伟目标。

今后五年的预期目标是：地区生产总值年均增长9%，规模以上工业增加值年均增长11%，全社会固定资产投资年均增长16%，社会消费品零售总额年均增长12%，地方一般公共预算收入年均增长10%，城镇居民人均可支配收入年均增长10%，农村居民人均可支配收入年均增长11%。

今后五年的主要任务是：

——率先实现次级突破。坚持稳中求进、进中求好、好中求快，始终保持强劲发展势头，在全省率先实现次级突破。到2021年，地区生产总值达到2200亿元，地方一般公共预算收入达到220亿元，社会消费品零售总额达到1120亿元，累计完成全社会固定资产投资1万亿元。

——建成长江上游产业转型升级示范区。推进供给侧结构性改革，增强发展动力。围绕产业链布局创新链，加快传统产业转型升级，积极培育壮大新兴产业，促进产业迈上中高端。到2021年，力争新兴产业增加值占规模以上工业增加值的比重达到25%，工业对经济增长的贡献率达到58%，服务业比重提高5个百分点，现代农业实现新突破。

——建成成渝城市群南部区域中心城市。全面推进新型城镇化，完善城镇发展体系，提升城市服务功能，切实增强城市综合承载能力。智慧城市基本建成，城市品质和市民素质显著提升，如期实现“双两百”大城市目标。

——建成全国重要区域性综合交通枢纽。大力实施对外通道、市域交通网络建设，推动高铁、城市轻轨突破发展，加快高等级、广覆盖公路网建设，建成川滇黔航运物流中心，打造区域航空运输中心和国际航空港。到2021年，公路里程达到1.5万公里，集装箱年吞吐量达到100万标箱，泸州机场旅客年吞吐量达到200万人次。

——建成川滇黔渝结合部对外开放门户。深化区域合作，拓展国际国内市场。依托港口优势，主动融入沿江协同开放，建成成渝城市群南向开放、辐射滇黔的重要门户。优化投资环境，着力招大引强，招引一批大企业、大项目落户泸州，到位市外国内资金年均增长15%以上。

——建成幸福美丽泸州。完善社会保障体系，加快社会事业发展，提高社会文明程度和人民健康水平，建成全国文明城市和健康城市。坚持依法行政，基本建成法治政府。加快绿色发展，推进生态文明建设。到2021年，森林覆盖率达到51%，万元GDP综合能耗累计下降12%。坚决打赢脱贫攻坚战，到2019年全面消除绝对贫困。

各位代表，未来五年，任务艰巨、责任重大、使命光荣。我们坚信，只要干在实处、走在前列，“决胜全面小康、建成区域中心”的目标就一定能够实现！

2017年重点工作

2017年是新一届政府工作的开局之年，是实施“十三五”规划承前启后的关键一年。全市经济社会发展的主要预期目标是：地区生产总值比2016年（下同）增长9%，规模以上工业增加值增长11%，全社会固定资产投资增长16%，社会消费品零售总额增长12%，地方一般公共预算收入增长9%，城镇居民人均可支配收入增长9%，农村居民人均可支配收入增长10%。

围绕上述目标，重点抓好以下工作：

一、着力强化产业支撑，加快产业结构优化升级

优化提升现代农业。全力推进国家现代农业示范区建设，加快发展精品果业、现代养殖业、高效林竹业等“八大特色农业产业”。严守耕地红线，耕地保有量达到38.962万公顷。打造现代农业产业融合示范园15个。大力培育和引进现代农业经营主体，新增市级以上重点龙头企业20家、农民合作社示范社30家、家庭农场示范场20个。开展畜禽养殖标准化示范创建，推进温氏100万头优质生猪一体化、古蔺优质肉牛全产业链项目建设。加快泸县三星桥水库、古蔺石梁子水库等重点水利工程建设，新增有效灌面3.5万亩。

加快发展现代制造业。推进传统产业提质增效，加快泸州老窖酿酒工程技改、郎酒泸州包装基地扩建、聚碳酸酯（一期）等项目建设，开工建设酒业园区酱酒园,力争开工建设中海（四川）科技产业园，确保新火炬化工技改等项目竣工投产。培育壮大高端装备制造、现代医药、智能电网、新能源汽车、新材料、电子信息等新兴产业。加快步长生物制药基地、西南智能电网产业园、汽车科技产业园、电子信息产业基地建设，推进江麓容大无级自动变速器、中航EPS转向系统等汽车配套产业项目建设，确保华为大数据中心、许继电气等项目建成投用。

大力发展现代服务业。加快万达广场购物中心、西南商贸城（三期）、泸州金融商业中心等项目建设，积极培育城市新商圈。推进和润集团粮食物流及深加工、海吉星农产品国际商贸物流园、中国物流泸州配送物流园等项目建设，着力打造川南大宗商品交易市场。推进泸州电商产业园建设，电子商务交易额增长30%。泸州机场新开通1至2条航线，旅客吞吐量突破100万人次。泸州港集装箱吞吐量达到55万标箱、增长15%。加快发展会展经济，办好“三大展会”活动。支持银行拓展业务、做强做大，加快发展保险、证券等金融服务业。积极发展健康服务业，加快5个健康养老服务项目建设。大力发展文化旅游、生态旅游、乡村民俗旅游，推动黄荆、方山、天仙硐、画稿溪等景区提档升级，加快福宝国际旅游度假区、沙湾古镇旅游综合体等项目建设，启动酒镇·酒庄—凤凰湖国家5A级景区创建工作，实现旅游总收入400亿元。加大政策支持力度，促进房地产和建筑业平稳健康发展。

强化园区平台建设。泸州高新区围绕“高、新、快、特”，增强承载能力，完成投资100亿元，新增建成区面积5平方公里，主营业务收入增长20%，建成国家创新型特色园区。泸州长开区突出发展“六型经济”，完成投资80亿元，新增建成区面积4平方公里，主营业务收入增长25%，争创国家级经济开发区。酒业园区坚持“三产联动、产城融合”，做强做大主导产业，完成投资30亿元，新增建成区面积2平方公里，主营业务收入增长18%，争创省级经济开发区。加快建设空港产业园、军民融合产业园。全市产业园区实现主营业务收入2240亿元、增长15%。

二、强力实施项目攻坚，坚定不移推进投资拉动

着力优化投资结构。准确把握国家和省上投资方向，提高投资有效性和精准性，完成全社会固定资产投资2000亿元以上。积极拓宽社会资本投资领域，实施市本级PPP项目18个、完成投资200亿元。大力促进民间投资，力争实现民间投资1060亿元。突出产业投资，完成工业投资575亿元。

着力加快项目建设。认真落实“项目投资攻坚年”工作部署，实施重点项目609个，完成投资1547.7亿元、增长18.3%。其中：竣工投用项目82个、完成投资139.1亿元，加快建设项目178个、完成投资889.5亿元，新开工建设项目192个、完成投资519.1亿元，做好157个项目前期工作。突出抓好交通建设“大会战”，推进叙古高速古蔺至川黔界、G8515泸渝高速、叙威高速公路建设，力争开工建设泸永高速公路；加快川南城际铁路自贡至泸州段、隆黄铁路叙永至镇雄段等项目建设。

着力强化要素保障。促进投融资公司做强做大，拓宽融资渠道，增强融资能力。加强银政企合作，贷款增长15%以上。用好用活土地政策，提高土地利用效率，全面保障重点项目用地需求。扩大直购电规模和范围，确保油、气、运等要素供给。

三、扎实推进新型城镇化，加快建设区域中心城市

加快中心城区建设。开展城市总体规划修编，完善主体功能区规划。强力推进“两江新城”建设，推动纳溪区与江阳区无缝对接、错位发展，推动泸县撤县设区、向南拓展。加快过江通道建设，确保沱江二桥加宽改造工程、沱江六桥建成通车，推进长江二桥、长江六桥建设。畅通城市干线、环线路网，推进交通拥堵节点改造，加快建设二环路，完成沱江新城南滨路、北滨路主体工程。启动新城新区地下综合管廊建设，加快实施老城区管网改造，增强城市防洪抗涝能力。建成投用茜草二水厂（一期）、主城区第二批停车场（一期），加快长江生态湿地公园、城西客运站、滨江路小关门至柏木溪段等项目建设。中心城区建成区面积新增18平方公里。

加快县城和小城镇建设。支持合江、叙永、古蔺加快县城发展，着力提升县城品质，确保合江城西湿地公园、叙永绕城路、古蔺民生广场等项目竣工投用，增强县城综合承载能力和吸纳能力。加快打造纳溪大渡口镇、合江九支镇、古蔺二郎镇、叙永江门镇等15个重点镇，全面启动特色小镇示范镇建设。

加快提升城市治理水平。推进老城区全域全面治理，完成老旧道路“白加黑”升级改造。加快城市治理立法进程，推进市本级、江阳、龙马潭、纳溪、泸县城市执法体制改革工作。扎实开展“三大专项整治”，分步推进城市建筑立面治理，营造清爽、整洁的城市环境。完善城乡环卫基础设施，加快建设城北垃圾压缩中转站，完成森泰垃圾处理场封场项目建设。创新举措，加大力度，力争建成全国文明城市。

四、深化改革扩大开放，不断增强发展动力活力

突出重点领域改革。深化“放管服”改革，动态调整行政许可项目，对保留行政许可事项实施标准化管理，推进合江、龙马潭相对集中行政许可权改革试点。深化财税体制改革，加快建立现代财政制度。加强债务管理，防范财政风险。深化国资国企改革，完善国有资产监管体制，推动市属国有企业做强做优做大，加快泸天化集团改革发展，力争华西证券、兴泸水务等企业上市。全面完成农村“七权”确权登记工作，加快推进农村土地制度改革，认真实施承包土地“三权分置”。加快农村集体资产股份合作制改革，推行“一改三化”，发展壮大集体经济，增加农民财产性收入。

深化对外开放合作。主动融入“一带一路”、长江经济带，推进深层次高水平双向开放。积极参与“251行动计划”，深化与东盟、俄罗斯、德国、韩国等重点区域和国家的贸易合作。加强与重庆、上海、杭州、惠州等城市务实合作，深化赤水河区域及毗邻地区合作发展。着力招大引强，瞄准重点产业、重点地区、重点企业进行精准招商，力争在工业招商，尤其是承接深圳电子信息产业转移方面取得突破，到位市外国内资金增长15%以上。

加快建设开放口岸。主动融入沿江沿海开放大格局，积极建设国家水运开放口岸。加快政策制度创新，协同推进多式联运与通关一体化建设，促进投资和贸易便利化。大力发展先进制造、航运物流、港口贸易、融资租赁等临港产业，促进食品饮料等优势产业走向国际市场。

五、大力发展县域经济，构建区县竞相发展格局

做强民营经济支撑。紧紧围绕振兴实体经济，加快培育市场主体，放宽民营企业市场准入，鼓励民间资本进入基础设施、公共事业等行业和领域。支持民营企业开展强强联合、兼并重组，促进民营企业做大做强，推动中小微企业梯队成长。用好用活资本市场，力争鑫霸实业、陈年窖等企业在“新三板”挂牌。强化联系帮扶，引导民营企业完善现代企业制度，着力构建“亲”“清”新型政商关系。力争民营经济增加值达到950亿元。

深入推进大众创业、万众创新。完善创业扶持政策，加强创新基地建设，积极培育创新工场、创客空间，引导高校毕业生、农业转移人口等自主创业，力争全市新增孵化器面积20万平方米，新创办企业5000户以上。大力实施国家高新技术企业倍增计划，新培育高新技术企业12家，实现高新技术产值230亿元、增长15%。加快国家知识产权试点城市建设，持续推进与高校、科研院所产学研合作，新建一批具有本地特色的科研院所、创新战略联盟和标准化技术组织。实施品牌质量塑造行动，加强泸州品牌培育提升和营销推广，形成品牌竞争新优势。

优化县域经济发展环境。加强政策支持，完善评价考核和激励机制，优化区县财权、事权配置，激发县域经济发展活力。大力实施扩权强镇，优先支持重点集镇基础设施和公共设施建设，促进重点集镇发展壮大。坚持全市“一盘棋”，加强产业发展、招商引资、基础设施建设、投融资等方面的合作，实现区县、园区互动发展。

六、全力推进精准扶贫，坚决打赢脱贫攻坚战

全面实施“四个一批”脱贫计划。紧紧围绕“两不愁三保障”和“四个好”目标，加快脱贫攻坚步伐。加大产业到户扶持力度，加快发展种养业、旅游、农村电商等特色产业，扶持生产和就业发展3.9万人。搬迁安置5.6万人，提前完成易地扶贫搬迁任务。深入实施医疗卫生精准扶贫“八百工程”，确保建档立卡贫困人口县域内医疗“零自付”。全面落实教育扶贫各项政策，确保贫困家庭学生不因贫失学。继续实施低保线、脱贫线“两线合一”，切实做到应保尽保。确保82989名农村贫困人口脱贫、149个贫困村退出、合江县脱贫“摘帽”。

深入推进“七大扶贫攻坚行动”。推进贫困地区交通、水利、电力、通信等基础设施建设，新改建贫困村通村水泥路（油路）273公里，完成赤水河环线扶贫公路建设，建成“渡改桥”8座，全面完成贫困村电网改造升级，提升“宽带乡村”服务能力。加强村级党群服务中心、卫生室、文化室等公共服务设施建设，提升贫困村公共服务水平。大力开展“四好村”创建活动，实施农户“三建四改”工程，让贫困地区广大群众住上好房子、过上好日子、养成好习惯、形成好风气。

建立完善脱贫攻坚工作机制。做好对口定点扶贫工作，完善“五个一”全覆盖机制。健全资产收益扶贫机制，推动资源变资产、资金变股金、农民变股东。创新金融扶贫机制，大力发展扶贫小额信贷，深入开展农村产权抵押融资试点。创新社会扶贫机制，吸纳更多社会资金投入扶贫。建立成果巩固机制，增强脱贫群众增收能力。

七、加快发展社会事业，切实保障和改善民生

扎实推进民生工程。确保公共财政用于民生支出的比重保持在70%以上。加强重点人群就业服务，城镇新增就业3.6万人，城镇登记失业率控制在4%以内。积极实施全民参保登记计划，完善城乡居民大病医疗保险办法。健全社会救助体系。加快保障性安居工程建设，实施棚户区改造9000户、农村危房改造1.3万户。加快养老服务体系建设，新增养老床位2500张，新建城乡社区日间照料中心89个。做好气象、水文监测预报，强化地质灾害预警防治，完成739户避险搬迁安置、2处重大地灾工程治理。

大力发展社会事业。推进义务教育均衡发展，加快发展普惠性学前教育和职业教育，新建公办中小学校15所、主城区公办幼儿园6所，加快西南医科大学博士学位授予点申报工作。持续推进医药卫生体制改革。加快医疗卫生服务体系建设，确保市人民医院创建为“三甲”医院，推进西南医疗康健中心（二期）、市中医院新院等项目建设，新改建中心乡镇卫生院23个、中心村卫生室232所。深入推进全民预防保健服务体系建设，加快建设健康城市。大力发展文体广电事业，新建一批文化体育设施，加强文化遗产保护，强化泸州历史文化名城的保护和发展。深入开展文化惠民活动、全民健身活动，努力扩大文化消费。

加快生态文明建设。强化资源节约集约利用，全力推进总量减排，加快淘汰落后产能，完成省上下达的节能减排任务。扎实抓好大气、水、土壤污染防治，强化大气污染联防联控，持续改善环境空气质量；大力开展“两江四河”重点流域污染整治，全市国控考核断面水质优良比例达到71.4%，县级以上城市集中式饮用水水源地水质达标率100%，建成投用乡镇污水处理厂46个。开展新一轮绿化泸州行动，继续推进天然林资源保护，实施新一轮退耕还林。综合治理水土流失面积180平方公里。

全面加强社会治理。深入开展“七五”普法工作。认真做好社会稳定风险评估，加强社会矛盾纠纷风险预警和排查化解。扎实开展信访专项整治，落实信访工作责任制，积极营造规范有序的信访环境。完善社会治安立体化防控体系，集中整治社会治安突出问题，严厉打击违法犯罪活动。严格落实安全生产主体责任、监管责任，持续开展“打非治违”和安全生产专项整治，坚决遏制重特大事故发生。强化食品药品和农产品质量安全监管，积极创建国家食品安全城市、省级农产品质量安全监管示范市。

加强国防动员和后备力量建设，积极发展妇女儿童、老龄和残疾人事业，切实做好对口援助、双拥、人民防空、民族宗教、外事、侨务、保密、统计、档案、地方志等工作。

八、加强政府自身建设，全面提升行政服务水平

深入推进法治政府建设。提高依法行政的意识和能力，依法全面履行政府职能。认真执行市人大及其常委会的决议、决定，主动接受市人大、市政协监督，切实提高办理人大代表建议、政协委员提案的质量和效率。严格执行重大行政决策程序规定，实行重大行政决策监察、审计、法制“三方会审”制度，落实重大行政决策责任追究。加强行政复议和行政应诉工作。完善政府立法体制机制，提高政府立法公众参与度。加强行政执法监督，规范行政执法裁量权。

深入推进效能政府建设。健全政务服务体系，积极推进县（区）、乡（镇、街道）、村（社区）“三级联动服务” ，不断提高政务服务的质量和效率。深化政务公开，坚持以公开为常态、不公开为例外，推进行政决策公开、执行公开、管理公开、服务公开和结果公开。积极探索“互联网+政务服务”，加强市级政务云平台建设和政府网站管理。深入推进行政权力依法规范公开运行，建立完善行政权力运行数据库，力争实现行政权力事项全业务全流程网上办理。规范公共资源交易管理。优化并联审批协调机制，创新事中事后监管，全面落实“双随机、一公开”制度。

深入推进廉洁政府建设。严格落实党风廉政建设责任制，把廉洁从政贯穿到政府工作各个环节。深入推进惩防体系建设，认真落实“三个在前”“三早三卡”，持之以恒正风肃纪，坚定不移惩治腐败。强化行政监察、审计监督和社会监督，加强重点领域、关键环节和重要岗位权力运行的监督管理，让权力在阳光下运行。

各位代表，新形势催人奋进，新征程再创辉煌！让我们紧密团结在以习近平同志为核心的党中央周围，在省委省政府和市委的坚强领导下，团结一心、锐意进取，为“决胜全面小康、建成区域中心”而努力奋斗!
